# Supplementary material for: Discovery of Synergistic Broadly Neutralizing Antibodies Targeting Non-Dominant Epitopes on SARS-CoV-2 RBD and NTD
Source: Vaccines (Basel). 2025 May 30;13(6):592. doi: 10.3390/vaccines13060592 (PMC12197520; doi:10.3390/vaccines13060592)
Supplement: Supplementary file 1 [file vaccines-13-00592-s001.zip › vaccines-3640171-supplementary.pdf]

vaccines

MDPI

Figures and figure legends

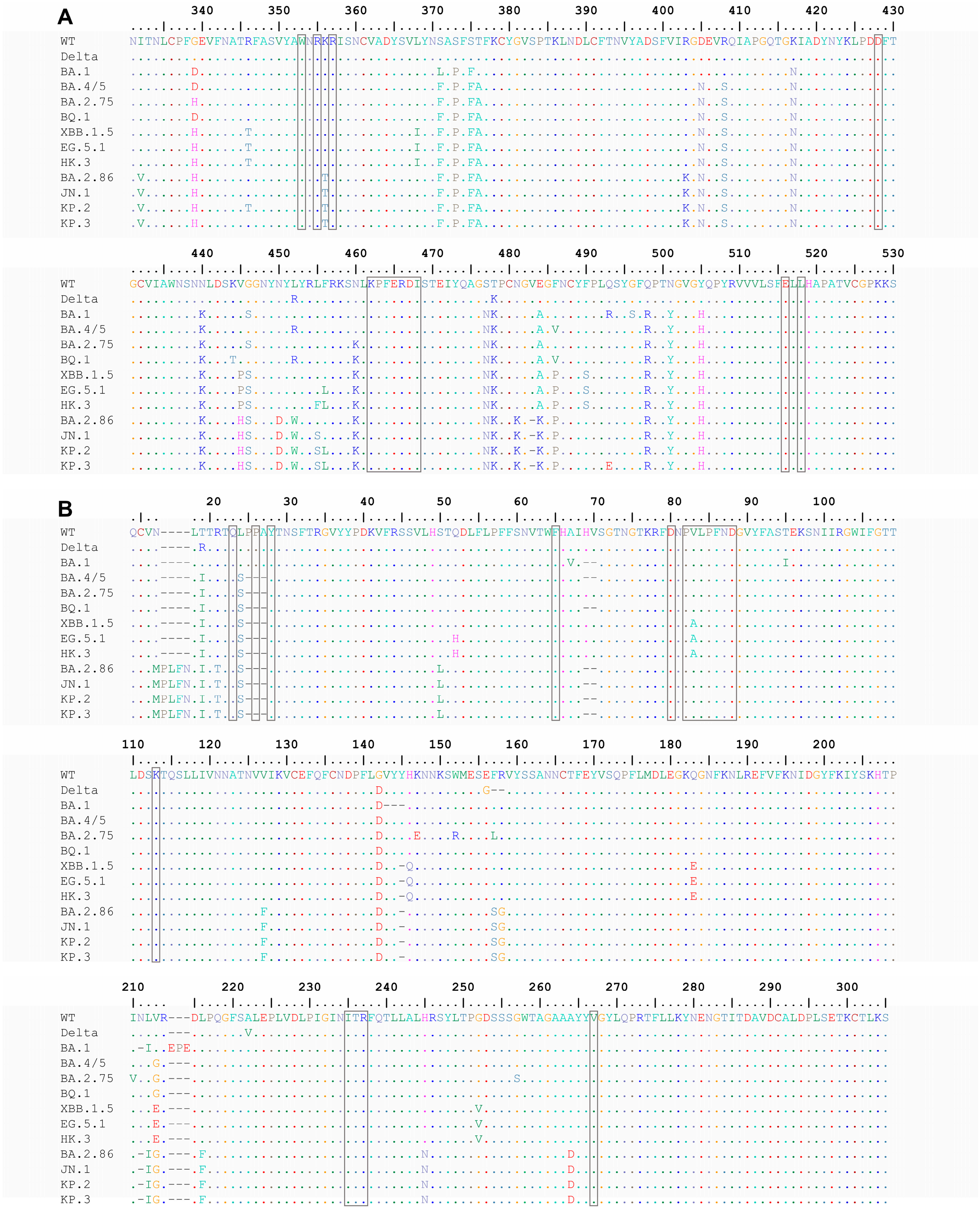

 https://doi.org/10.3390/vaccines13060592

Figure S1. Conservation of epitopes recognized by the four bnAbs. RBD (A) and NTD (B) sequences from all tested SARS-CoV-2 variants are aligned. The predicted epitopes are indicated by the boxes.

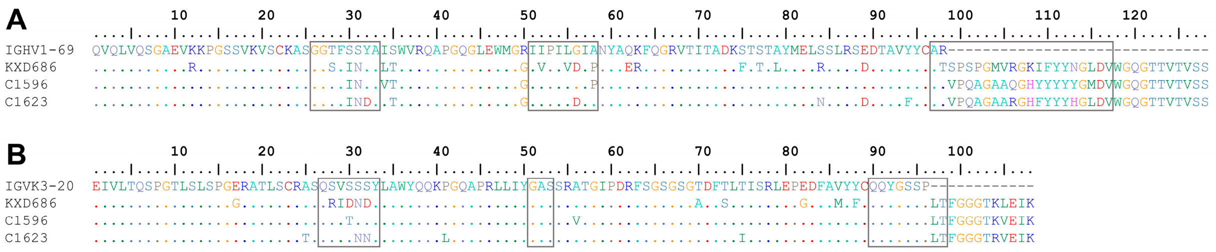

Figure S2. Sequence alignment of KXD686, C1596 and C1623. VHs (A) and Vκs (B) of KXD686, C1596 and C1623 are aligned with their corresponding V genes. CDRs are indicated by the boxes.
